# Supplementary material for: Activation of the hypothalamic feeding centre upon visual prey detection
Source: Nat Commun. 2017 Apr 20;8:15029. doi: 10.1038/ncomms15029 (PMC5411483; doi:10.1038/ncomms15029)
Supplement: Supplementary Information — Supplementary Figures [file ncomms15029-s11.pdf]

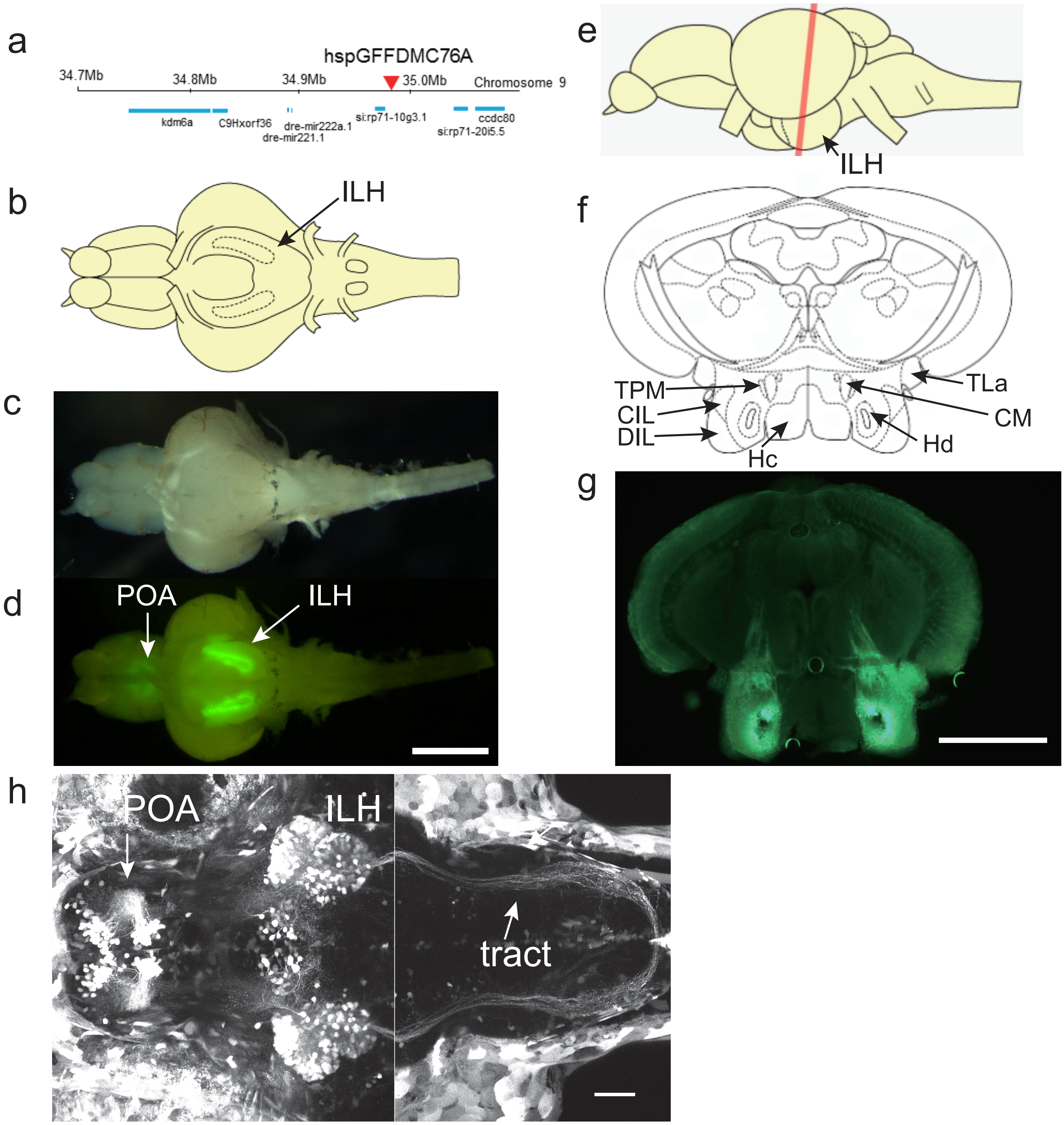

Supplementary Figure 1. UAS:EGFP reporter gene expression in the hspGFFDMC76A Gal4 line

a, Insertion site of the hspGFFDMC76A was identified in an intergenic region on chromosome 9. **b.** Schematic of a ventral view of the brain of an adult zebrafish. **c,** Ventral view of a dissected brain of an adult UAS:EGFP;hspGFFDMC76A fish. **d,** EGFP fluorescence in the brain shown in **b**. Scale bar: 1 mm. **e,** Schematic side view of the adult brain. The red line indicates the positions of the sections in **f** and **g. f,** Annotations based on Wulliman et al., 1996. **g,** EGFP fluorescence of a coronal section of the brain of an adult UAS:EGFP;hspGFFDMC76A zebrafish. Scale bar: 0.5 mm. **h,** UAS:EGFP expression in the hspGFFDMC76A Gal4 line at 5 dpf. Projected z-stack images obtained by two-photon laser microscopy. Left: z-stack projection of 155 slices (1 µm-step). Right: z-stack projection of 135 slices (1 µm-step). The positions of the focal planes of the two z-stacks overlap by 35 um (the right one more dorsal) along z-axis. Scale bar: 50 µm. CIL, central nucleus of the inferior lobe; CM, corpus mamillare; DIL, diffuse nucleus of the inferior lobe; Hc, caudal zone of periventricular hypothalamus; Hd, dorsal zone of periventricular hypothalamus; ILH, inferior lobes of the hypothalamus; POA, preoptic area; TLa, torus lateralis; TPM, tractus pretectomamillaris.

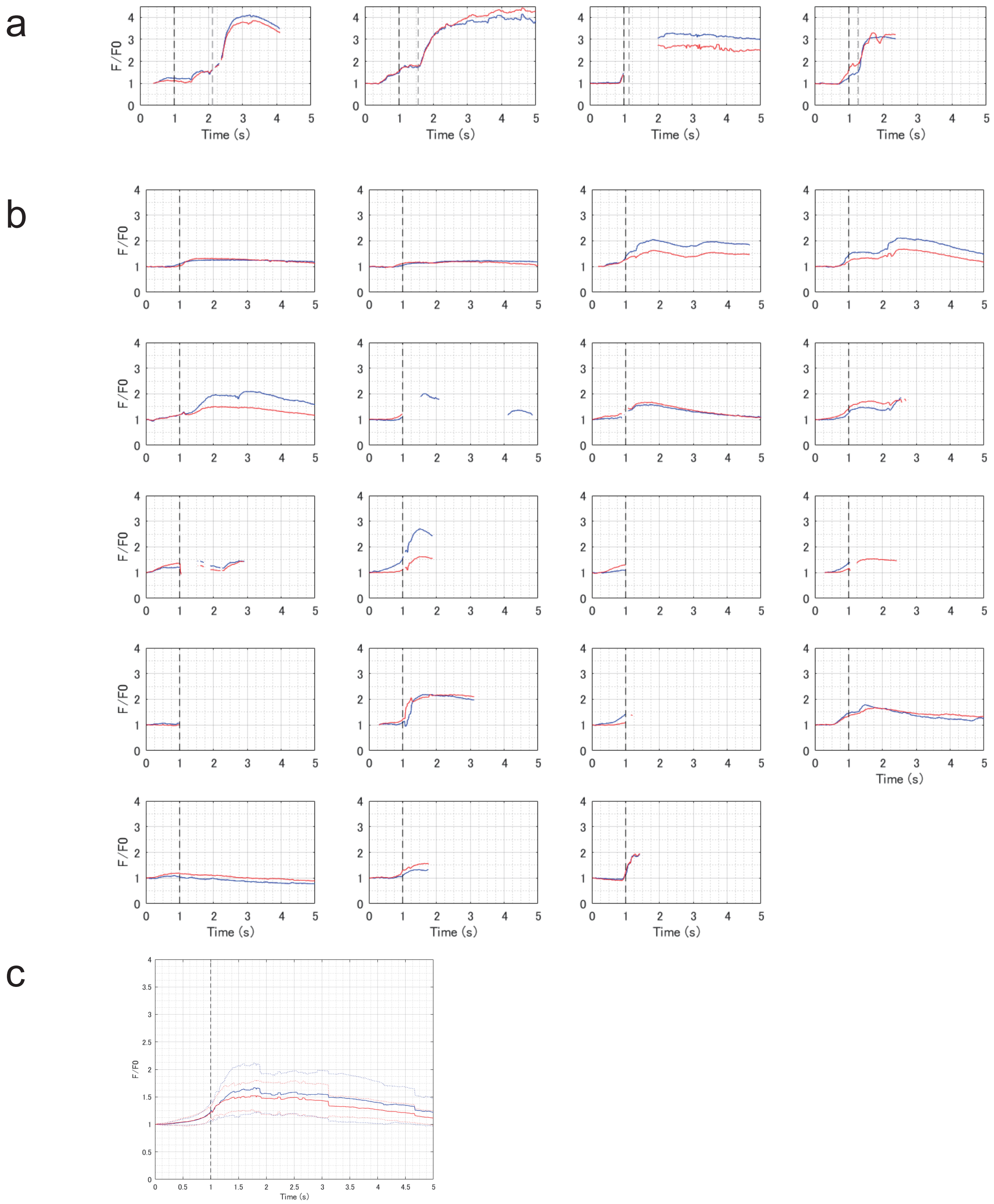

Supplementary Figure 2. Neuronal activity in the ILH in free-swimming larvae

a, Calcium signals in the left (blue) and right (red) ILH during prey capture behaviour in 4 dpf UAShspGCaMP6s;hspGFFDMC76A larvae. Four examples of successful prey capture events that were observed in 4 larvae (example on far left is also shown in Figure 1c). The black dotted vertical line represents the time of eye convergence. The grey dotted vertical line represents the time of the completion of the prey capture. The gaps in the graph indicate times when the image registration failed due to large/rapid movements of the larvae. **b,** Calcium signals in the ILH during prey capture behaviour that were aborted before capture (19 examples in 4 larvae). The black dotted vertical line represents the time of eye convergence. **c,** Average calcium signals shown in **b**. The graphs are aligned relative to the timing of eye convergence. The steps in the graph are artefacts from missing data. Note that the calcium signals starts to increase before eye convergence. The solid lines represent the mean, and the dotted lines represent the S.D.

a

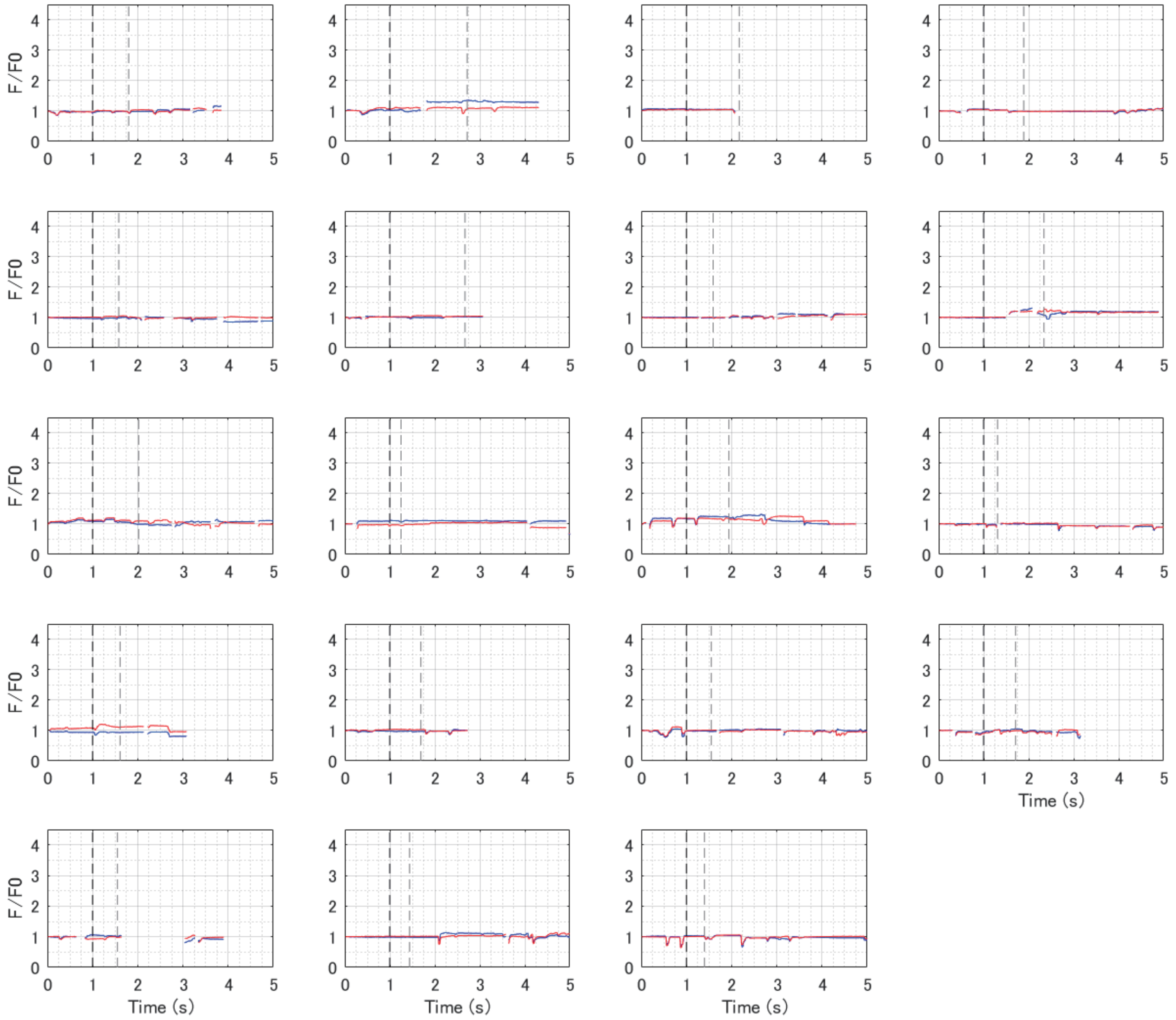

b

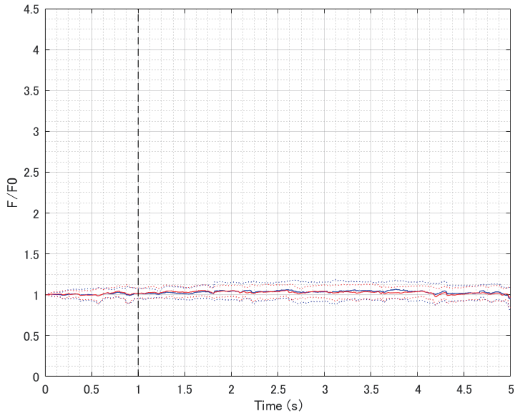

Supplementary Figure 3. EGFP fluorescence imaging in the ILH in free-swimming larvae

a, EGFP fluorescence signals in the left (blue) and right (red) ILH during prey capture behaviour in 5 dpf UAS:EGFP;hspGFFDMC76A larvae. Nineteen examples of successful prey capture events were observed in 3 larvae. The black dotted vertical line represents the time of eye convergence. The grey dotted vertical line represents the time of prey capture. The gaps in the graph indicate times when the image registration failed due to large/rapid movements of the larvae. **b,** Averaged EGFP fluorescence signals shown in **a**. The graphs are aligned relative to the timing of eye convergence. The solid lines represents the mean, and the dotted lines represent the S.D.

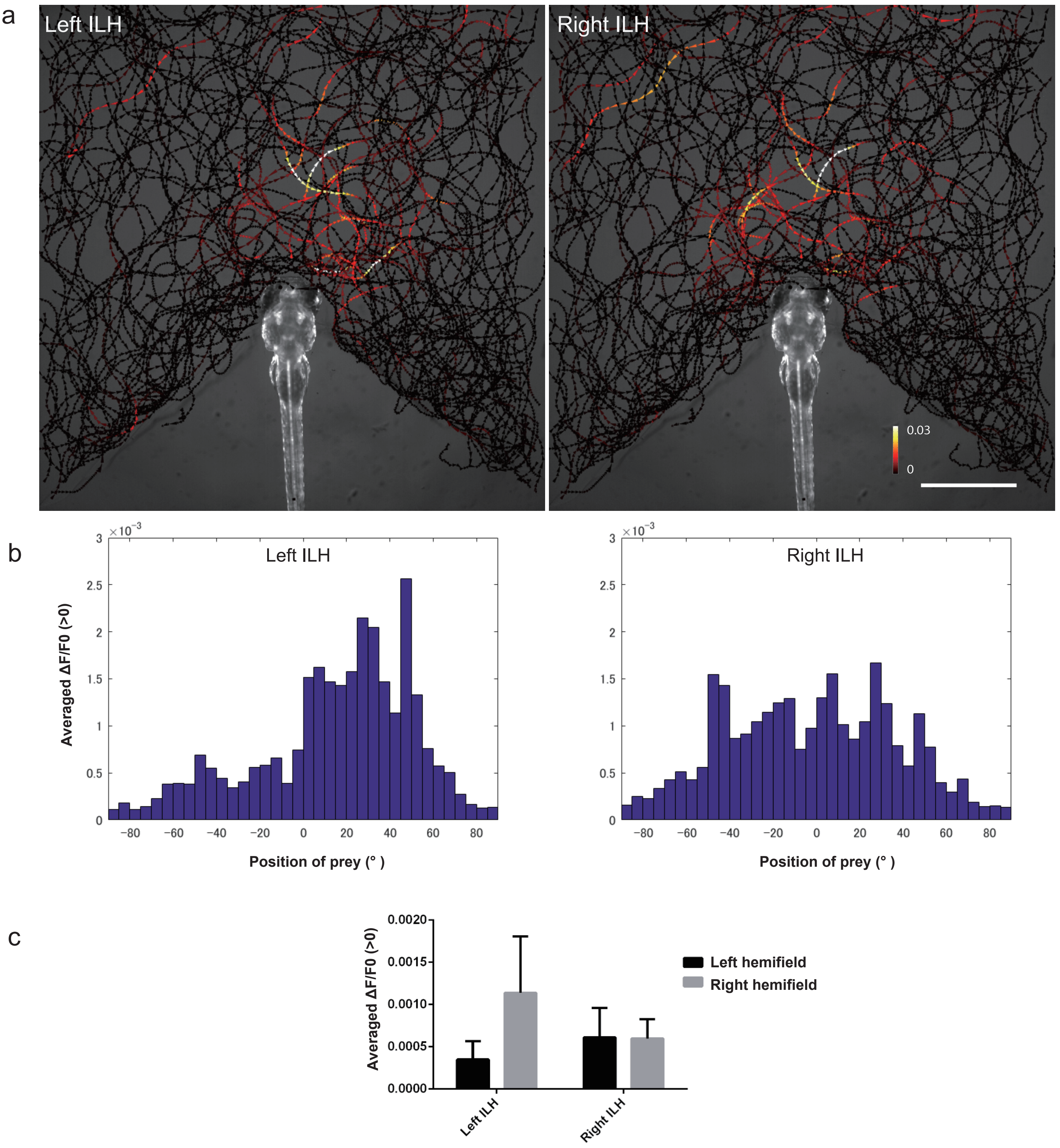

Supplementary Figure 4. Activity in the ILH at the sight of prey

a, The trajectories of single paramecia over 942 s are shown with the colour-coded changes in the intensity of GCaMP6s fluorescence in the left and right ILH areas in 5 dpf UAShspGCaMP6s;hspGFFDMC76A larvae. The data from 4 larvae were merged into a single larval image. Scale bar: 1 mm. The length of the arrowhead indicates the distance travelled by the paramecium in 60 ms. **b,** Average increase in the Ca signals in each 5° bin. The data are the same as those shown in **a**. **c,** Average increase in the Ca signals in the left and right hemifields. The data are the same as those shown in **a**. Mean ± S.D.

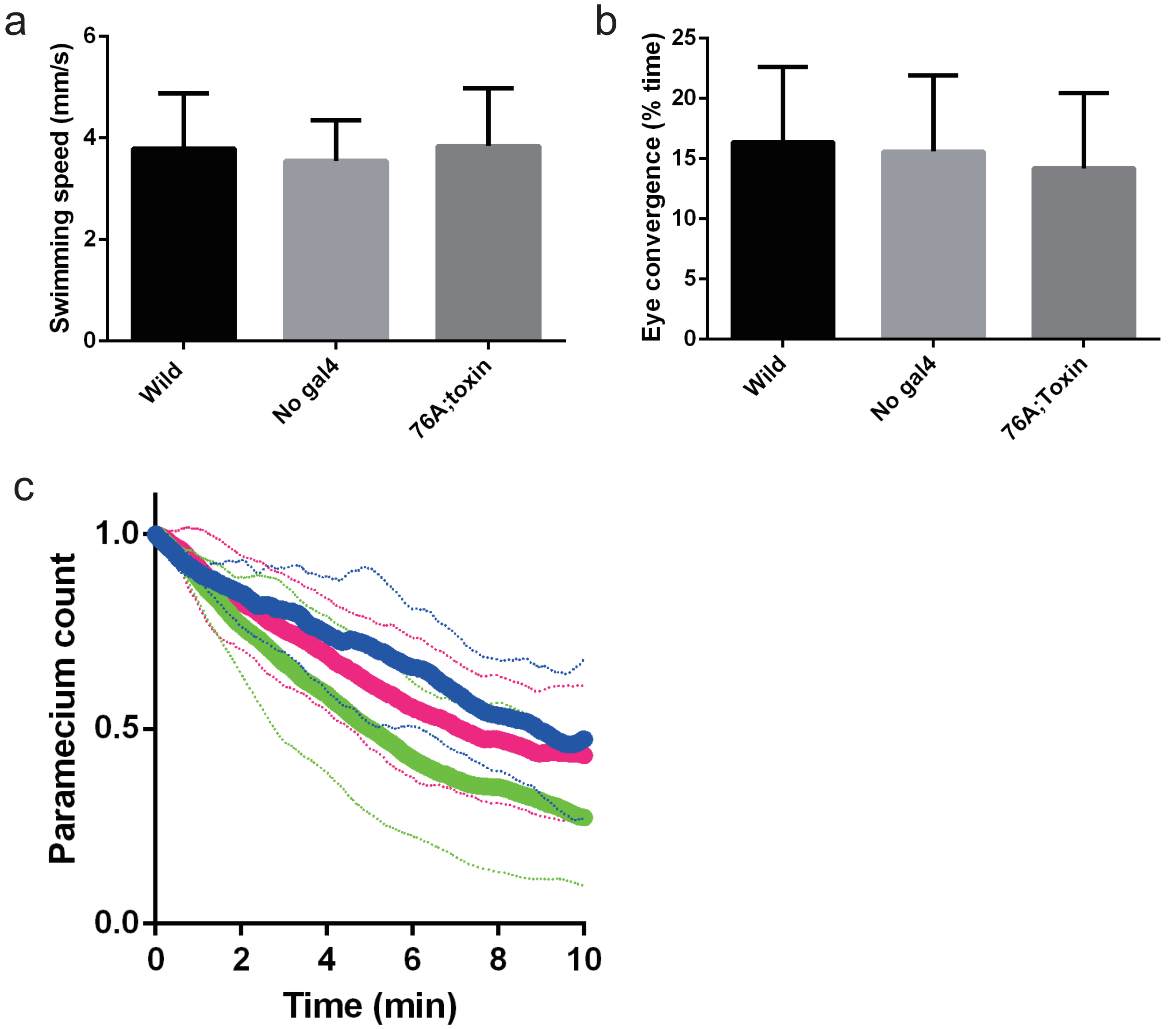

Supplementary Figure 5. Effects of botulinum toxin expression

a, Swimming speeds of wild-type (n=13), UAS:zBoTxBLCGFP (n=18), and UAS:zBoTxBLCGFP; hspGFFDMC76A larvae (n=6). Mean ± S.D. **b,** Eye convergence during 10 min with prey in wild-type (n=13), UAS:zBoTxBLCGFP (n=18), and UAS:zBoTxBLCGFP; hspGFFDMC76A larvae (n=6). Mean ± S.D. **c,** Paramecium consumption in 10 min in UAS:zBoTxBLCGFP;UAS:EGFP; SAGFF(LF)27A (magenta, n=9), UAS:zBoTxBLCGFP (blue, n=5) and UAS:EGFP;SAGFF(LF)27A (green, n=9) larvae. The solid lines represent the mean and the dotted lines represent the S.D. Two-tailed t-test between UAS:zBoTxBLCGFP and UAS:EGFP;SAGFF(LF)27A groups, p=0.07.

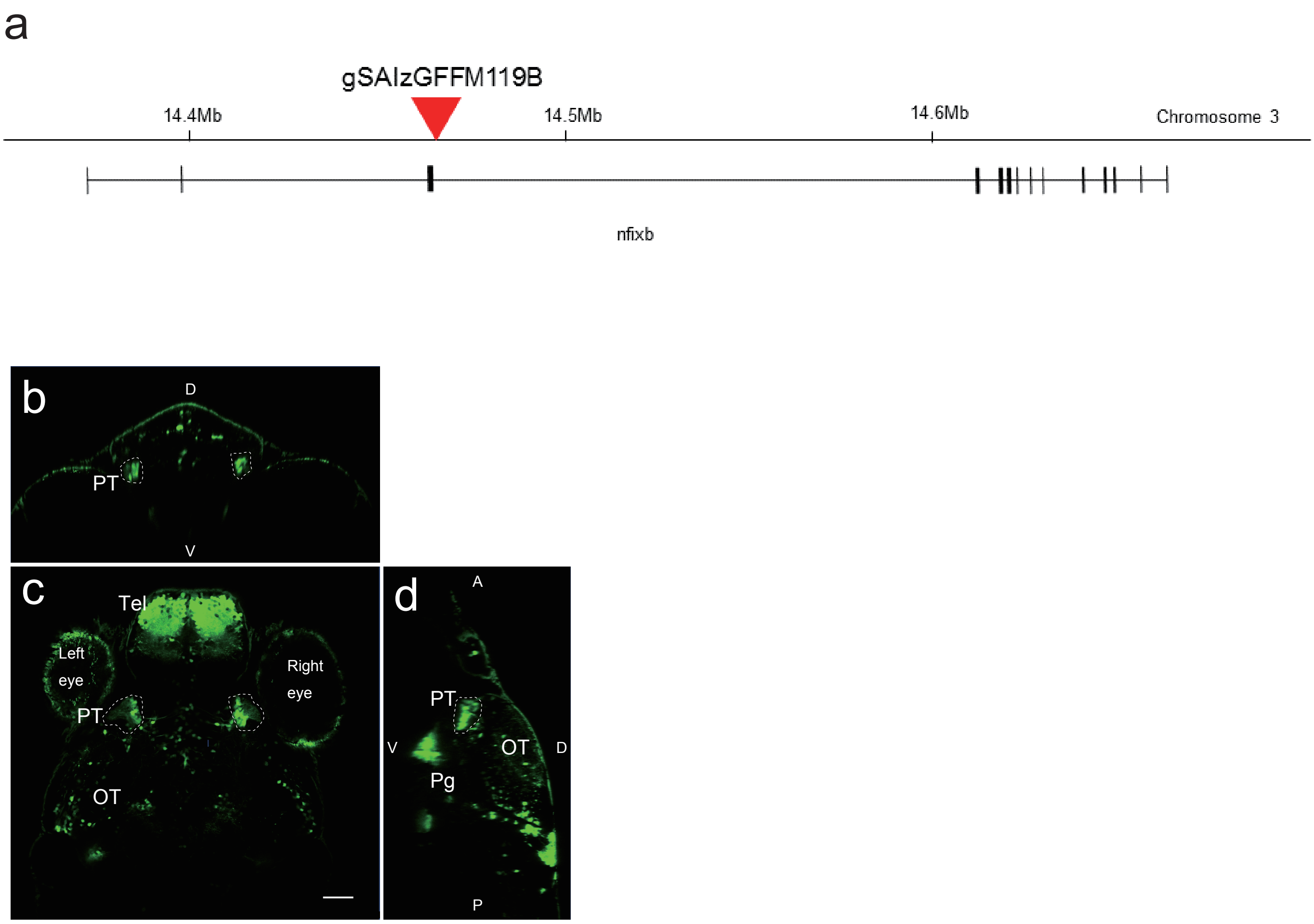

Supplementary Figure 6. The Gal4 insertion site in the gSAIzGFFM119B gene-trap Gal4 line and UAS:EGFP reporter gene expression

a, Insertion site of the Gal4 construct in the gSAIzGFFM119B line. The insertion site was identified within the *nfixb* gene on chromosome 3. **b-d,** Confocal images of the expression of the UAG:EGFP reporter, driven by Gal4 in the gSAzGFFM119B line. **b,** Coronal optical section. **c,** Dorsal view of a single optical section showing the positions of the pretectal cells. Scale bar: 50 µm. **d,** Sagittal view. D, dorsal; V, ventral; PT, pretectum; Tel, telencephalon; OT, optic tectum; Pg, preglomerular nuclei.

a

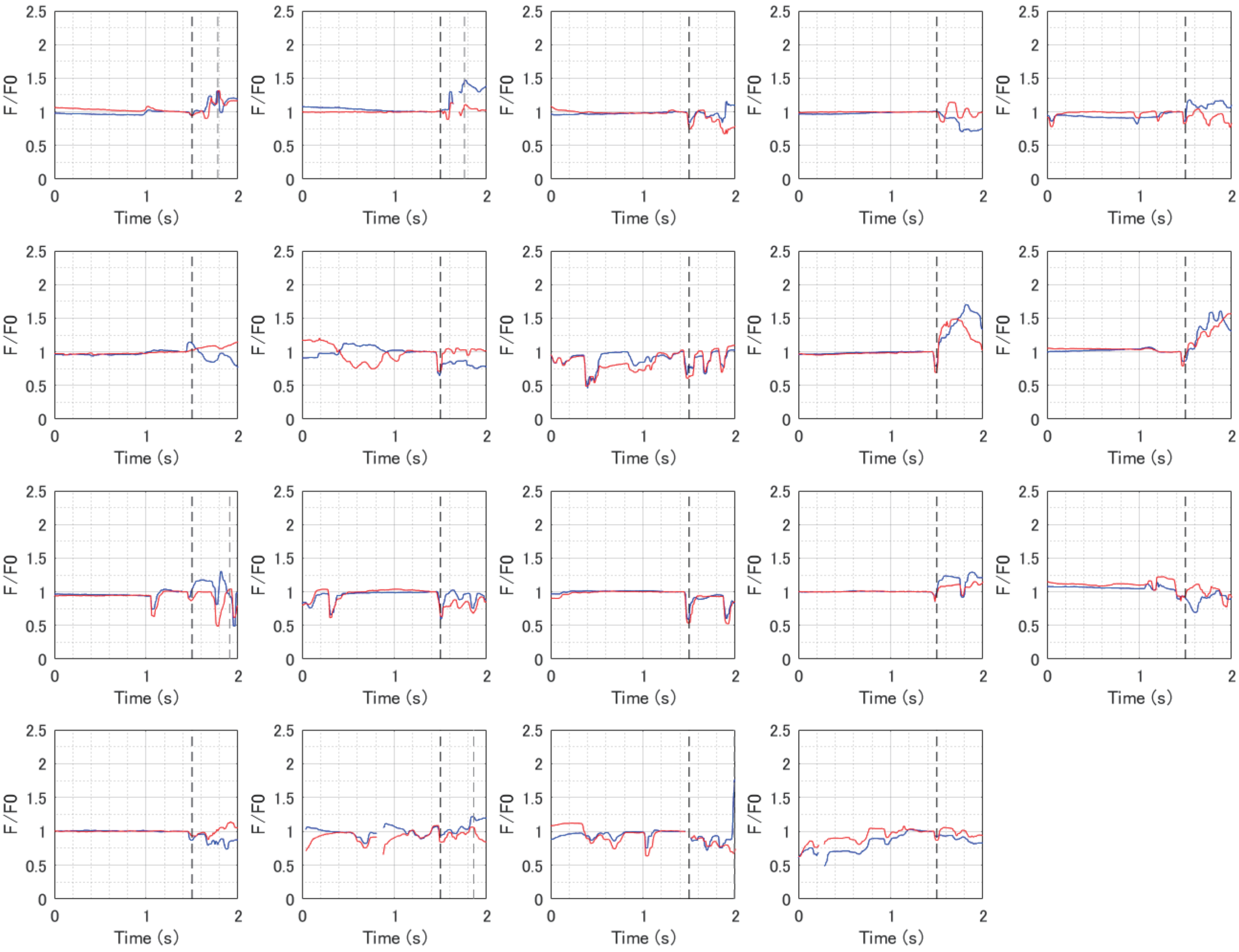

b

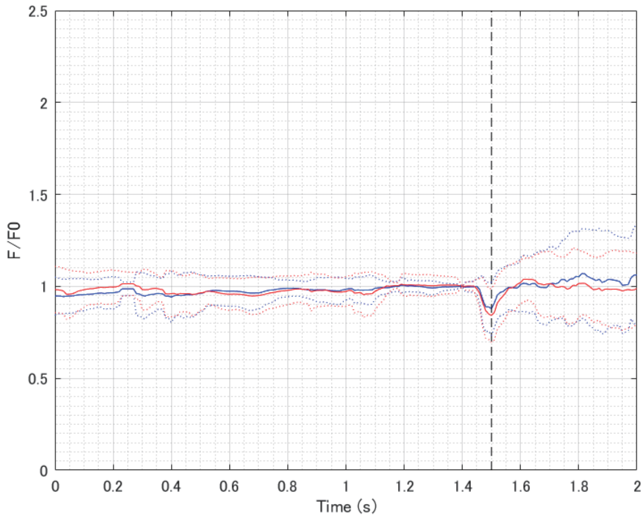

Supplementary Figure 7. EGFP fluorescence imaging in the pretectum in free-swimming larvae

a, EGFP fluorescence in the left (blue) and right (red) pretectum during prey capture behaviour in 5 dpf UAS:EGFP; gSAzGFFM119B larvae. The 19 examples of successful prey capture events that were observed in 3 larvae are shown. The black dotted vertical line represents the time of eye convergence. The grey dotted vertical line represents the time of prey capture (in some graphs, it occurred outside the time range). The gaps in the graph indicate times when the image registration failed due to large/rapid movements of the larvae. **b,** Averaged EGFP fluorescence signals shown in **a**. The graphs are aligned relative to the timing of eye convergence. The solid lines represent the mean, and the dotted lines represent the S.D.

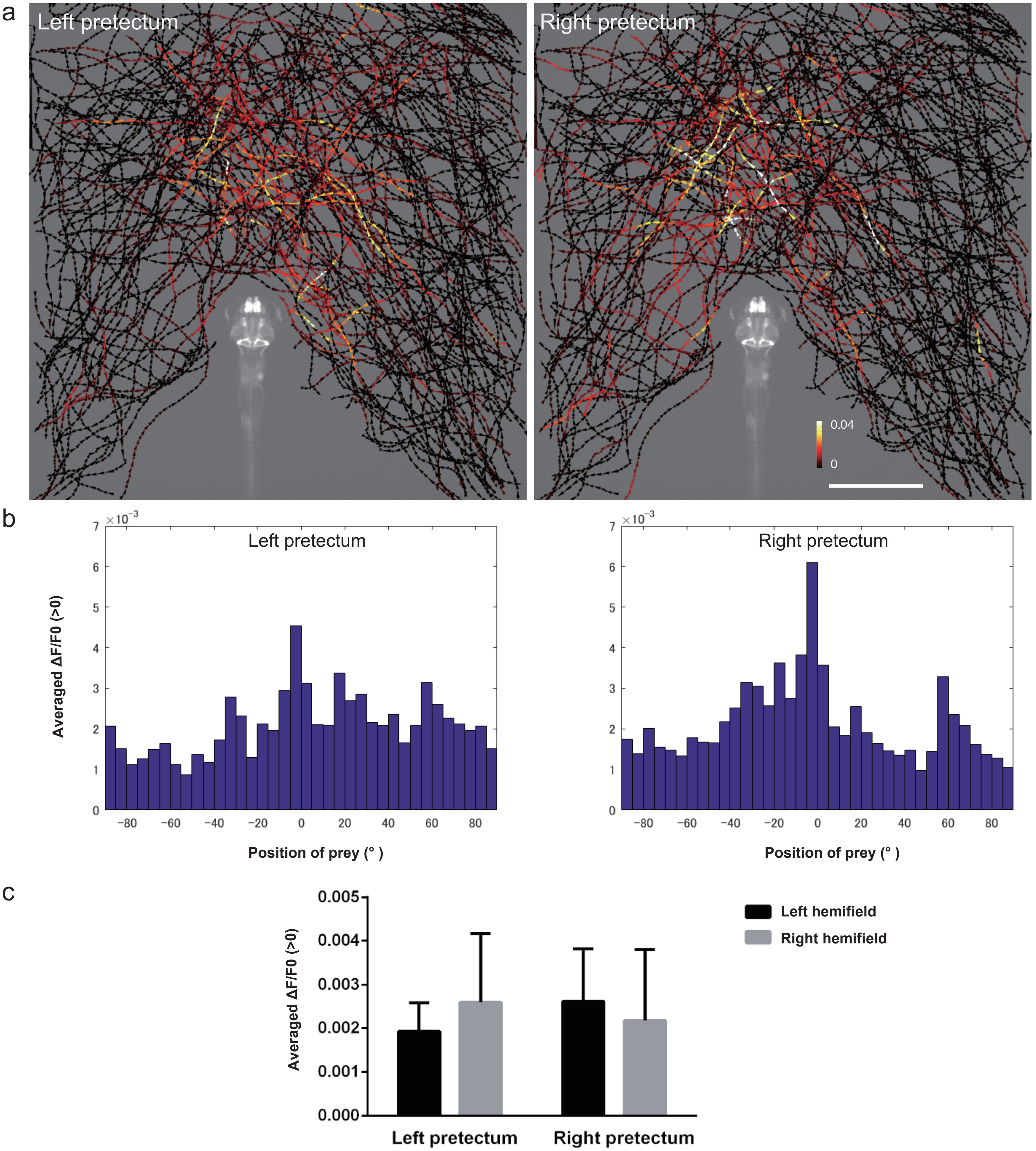

Supplementary Figure 8. Activity in the pretectal cells at the sight of prey

a, The trajectories of single paramecia over 775 s are shown with the colour-coded changes in the intensity of GCaMP6s fluorescence in the pretectum in 5 dpf UAShspGCaMP6s; gSAIzGFFM119B larvae. The data from 4 larvae are merged into a single larval image. Scale bar: 1 mm. The length of the arrowhead represents the distance travelled by the paramecium in 60 ms. **b,** Average increase in the Ca signals in each 5° bin. The data are the same as those shown in **a**. **c,** Averaged increase in the Ca signals in the left and right hemifields. The data are the same as those shown in **a**. Mean ± S.D.

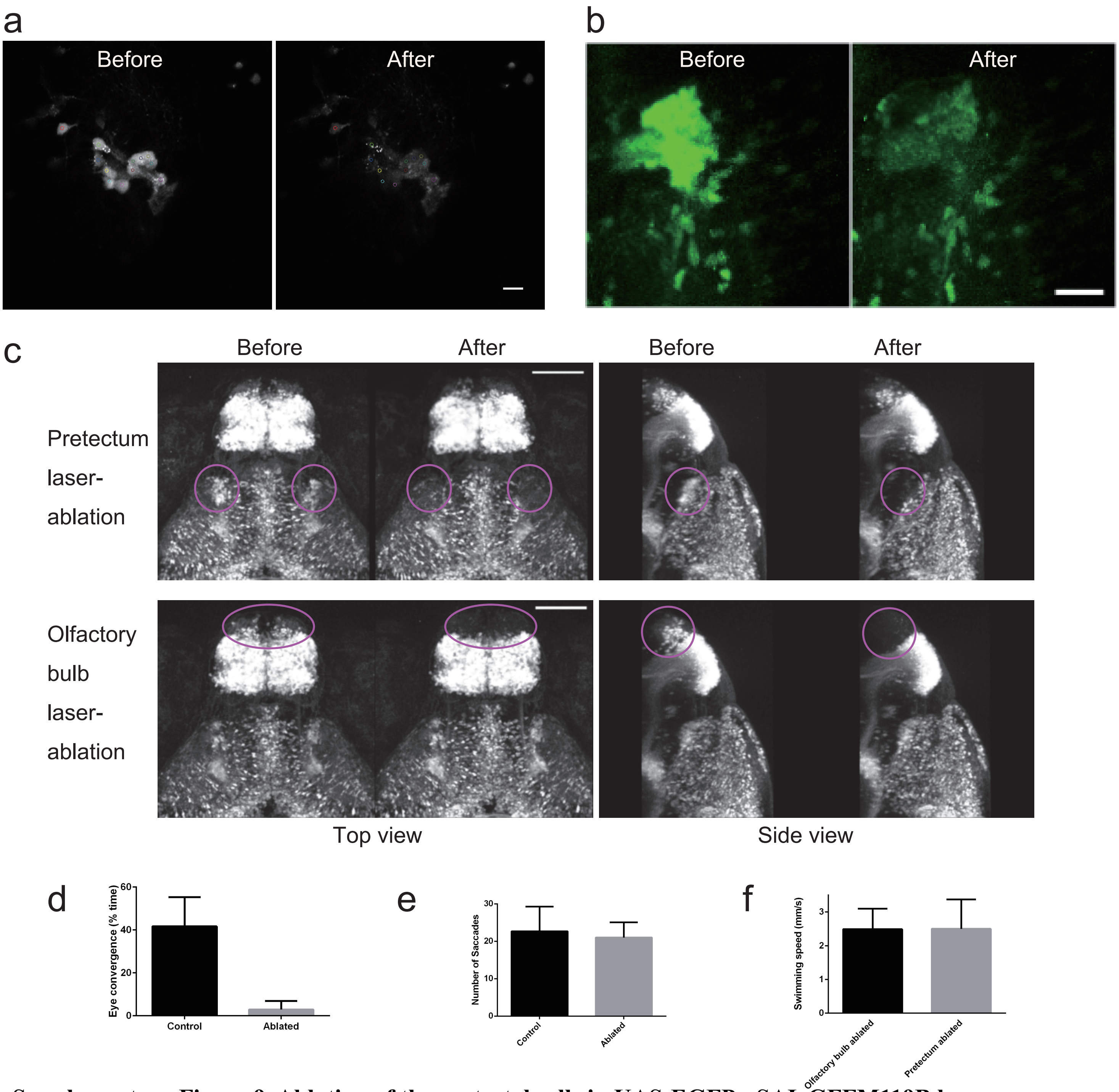

Supplementary Figure 9. Ablation of the pretectal cells in UAS:EGFP;gSAIzGFFM119B larvae

a, Images of the EGFP fluorescence of the pretectal cells on a focal plane before and after two-photon laser ablation in a 4 dpf UAS:EGFP;gSAIzGFFM119B larva. The targeted cells are labelled with small coloured circles. Scale bar: 10 μm.

b, Image of the EGFP fluorescence of the pretectal cells in a UAS:EGFP;gSAIzGFFM119B larva. z-stack images that covered the entire gregion of the 119B-labelled pretectal cells were projected onto one image. Before (left) and after (right) laser ablation. Scale bar: 25 μm. **c,** Absence of fluorescent cells after laser ablation. Top panel: pretectum ablation (encircled in magenta). Bottom panel: olfactory bulb ablation (encircled in magenta) as a control experiment. Scale bar: 100 μm. **d,** Eye convergence (indicator of prey capture, mean ± S.D.; Control, n = 3 untreated larvae; Ablated, n = 5 pretectum-ablated larvae; two-tailed t-test, $p = 0.0008$). **e,** Optokinetic response (mean ± S.D.; Control, n = 8 untreated larvae; Ablated, n = 6 pretectum-ablated larvae; two-tailed t-test $p = 0.5920$). **f,** Locomotor activity in olfactory bulb-ablated larvae (n=11) and pretectum-ablated larvae (n=10). The average swimming speeds in 10 min recordings are shown with S.D.

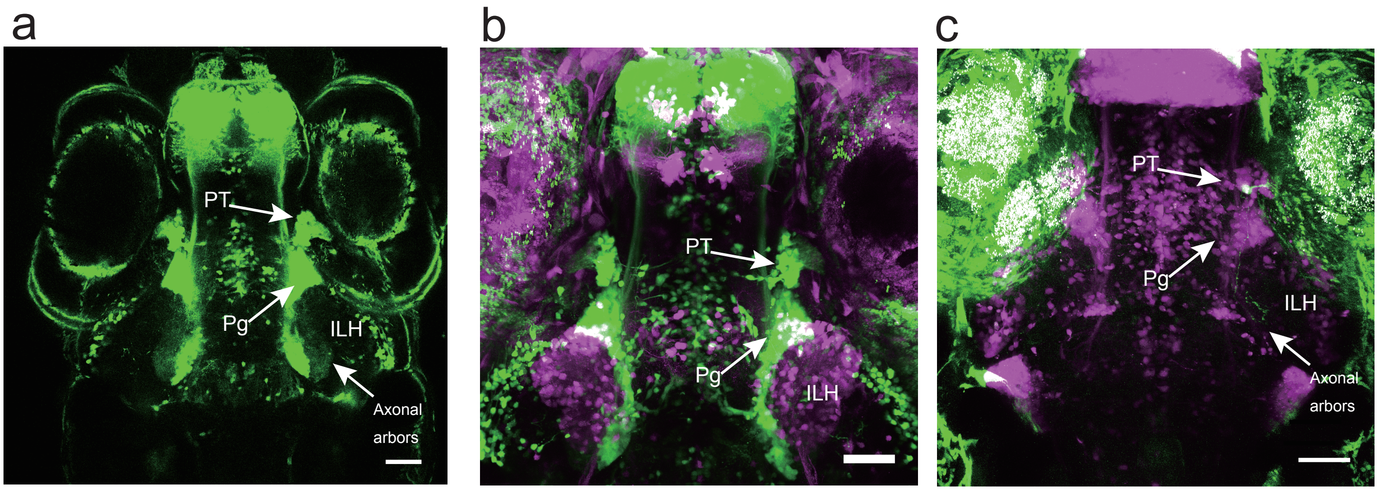

Supplementary Figure 10. Projection of the 119B-pretectal cells to the ILH

a, Merged image of 3 optical sections (66.7 μm and 22 μm apart on the Z-axis in the ventral direction) depicting the pretectal area (PT), the preglomerular nuclei (Pg), and the axonal arbours of the pretectal cells that reached the ILH at 5 dpf. Scale bar: 50 μm. **b,** Merged image of a UAS:EGFP;gSAIzGFFM119B larva (green) and a UAS:EGFP;hspGFFDMC76A larva (magenta, a different individual), which shows the location of the ILH relative to the PT and Pg. Scale bar: 50 μm. **c,** A single pretectal (PT) cell labelled by a UAS:EGFP DNA injection (green) into a UAS:RFP;gSAIzGFFM119B background zebrafish (magenta) at the 1-cell stage and observed at 4 dpf. An axon is extending out of the soma of the labelled cell in the pretectal area and is projecting to the caudomedial ILH. Scale bar: 50 μm.

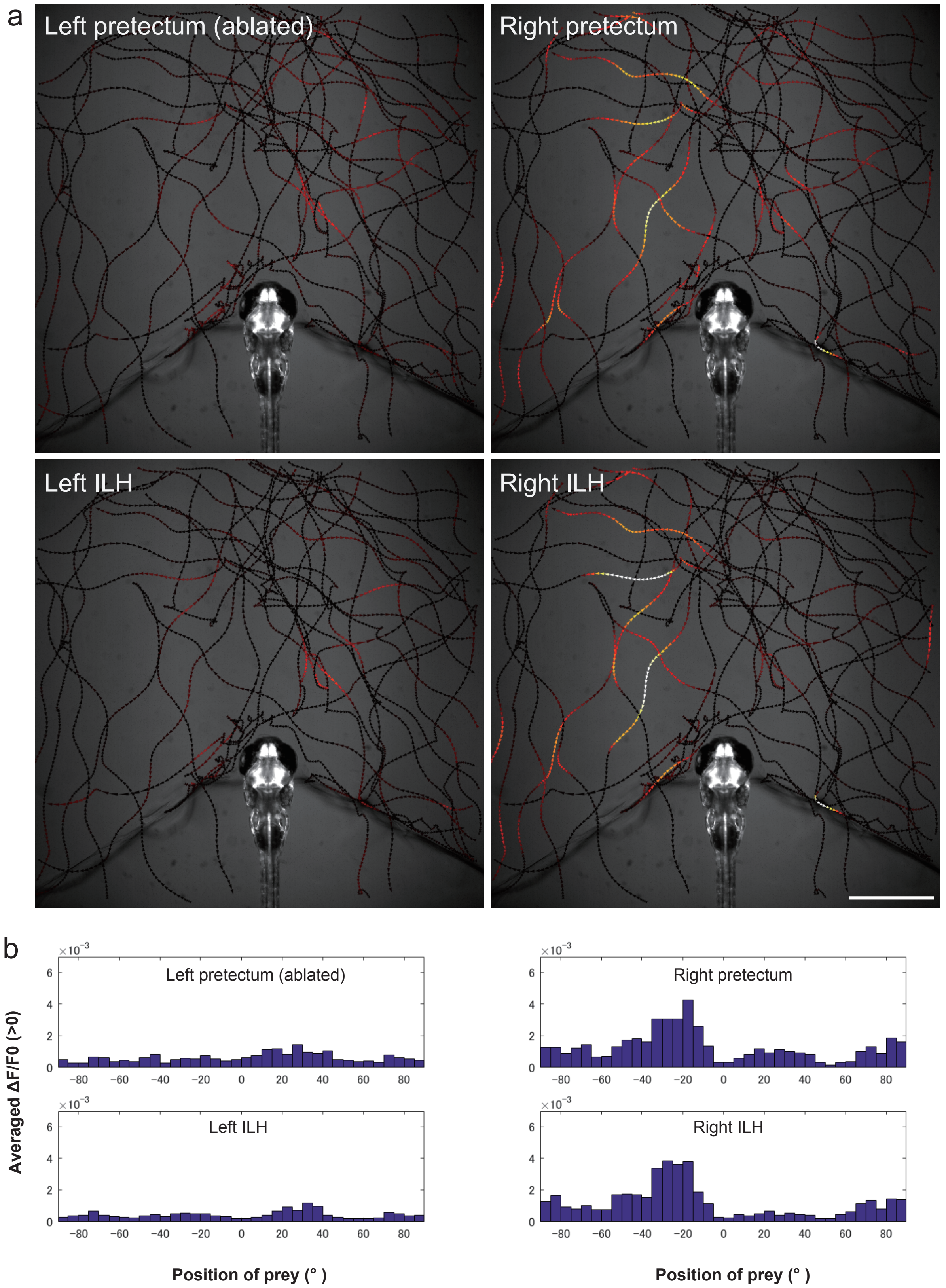

Supplementary Figure 11. Activity in the pretectum and ILH at the sight of prey in a larvae subjected to unilateral pretectum ablation

a, Another example of larvae in the unilateral ablation experiments shown in Figure 3c. The trajectories of a single paramecium over 250 s are shown with the colour-coded changes in the intensity of GCaMP6s fluorescence in a 5 dpf UAShspGCaMP6s; hspGFFDMC76A; gSAIzGFFM119B larva that was subjected to laser ablation of the left pretectum. Scale bar: 1 mm. The length of the arrowhead represents the distance travelled by the paramecium in 60 ms. **b,** Average increase in the Ca signals in each 5° bin. The data are the same as those shown in **a**.

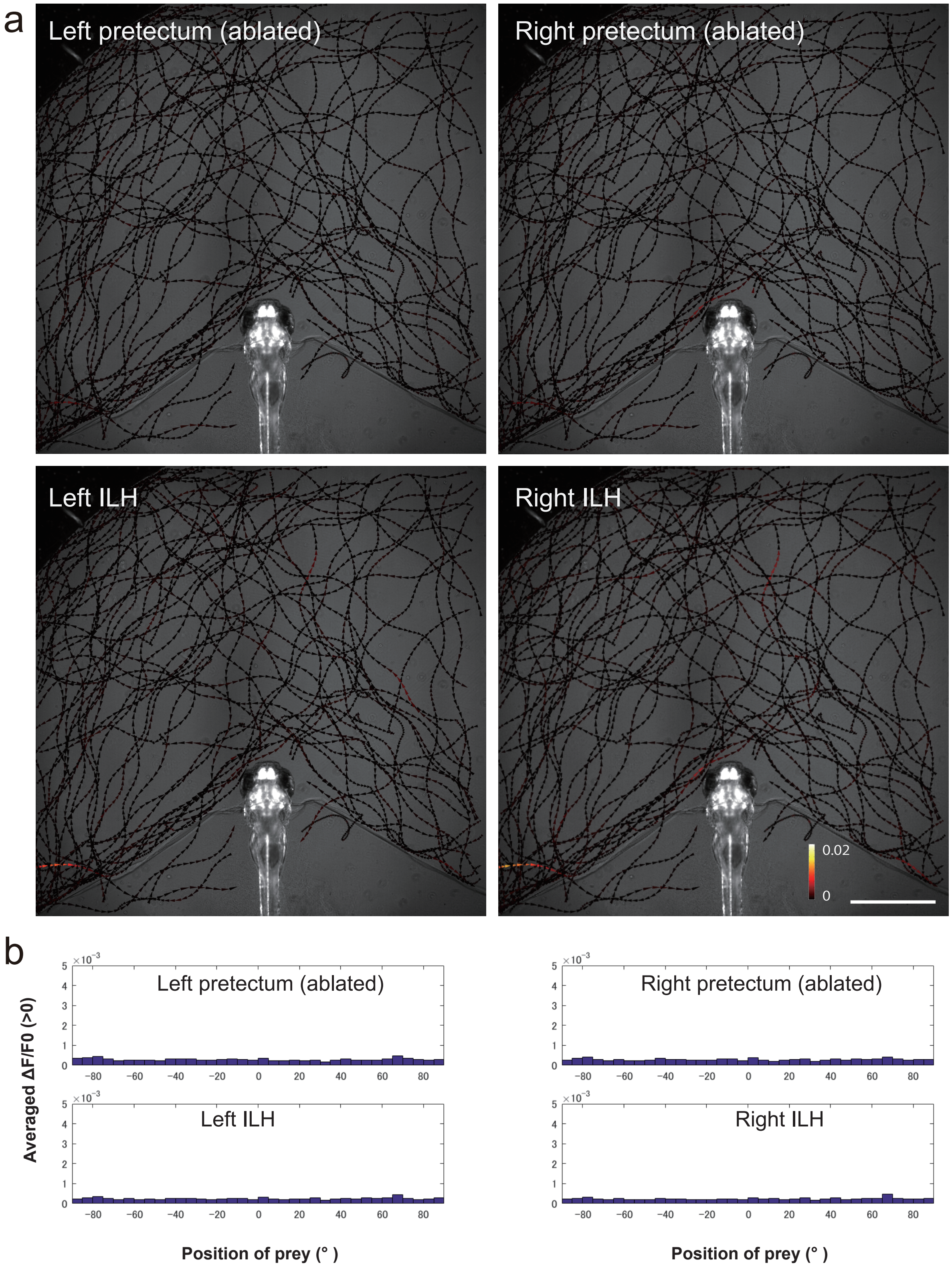

Supplementary Figure 12. Activity in the pretectum and ILH at the sight of prey in a larvae subjected to bilateral pretecutm ablation

a, A representative example of the larvae in the bilateral ablation experiments. The trajectories of single paramecia over 324 s are shown with the colour-coded changes in the intensity of GCaMP6s fluorescence in a 5 dpf UAShspGCaMP6s;hspGFFDMC76A; gSAIzGFFM119B larva that was subjected to laser ablation of the bilateral pretectum. Scale bar: 1 mm. The length of the arrowhead represents the distance travelled by the paramecium in 60 ms. **b,** Average increase in the Ca signals in each 5° bin. The data are the same as those shown in **a**.

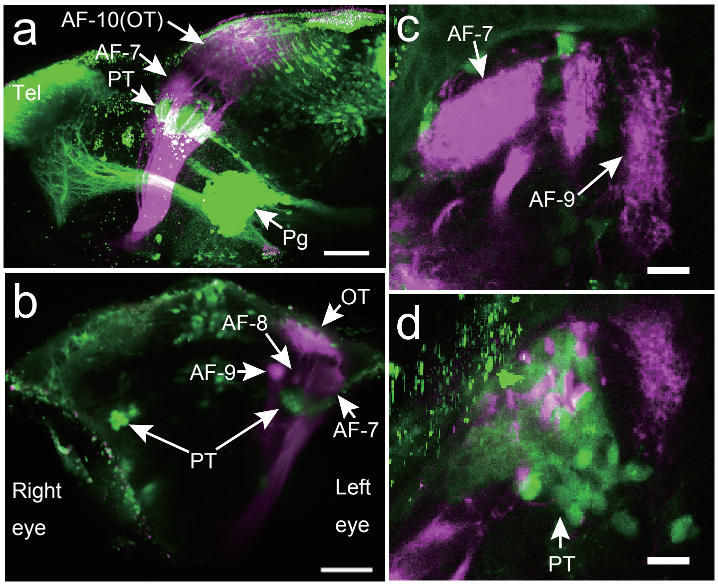

Supplementary Figure 13. Locations of the 119B-pretectal area and the retinal ganglion cell arbours

Locations of the axonal arbourisation fields (AFs) of the ganglion cells (magenta) relative to the gSAzGFFM119B-labelled cells (green). DiI was injected into the right eye, and the left eye was removed for observation. **a,** Lateral view from the left side. Scale bar: 50 µm. **b,** Front view. Scale bar: 50 µm. **c,** Top view (focused on the AF-7 area). Scale bar: 5 µm. **d,** Top view (focused 26 µm below the AF-7 area). Scale bar: 5 µm. PT, pretectal area; OT, optic tectum; Tel, telencephalon; Pg, preglomerular nuclei; D, dorsal; V, ventral; A, anterior; P, posterior.

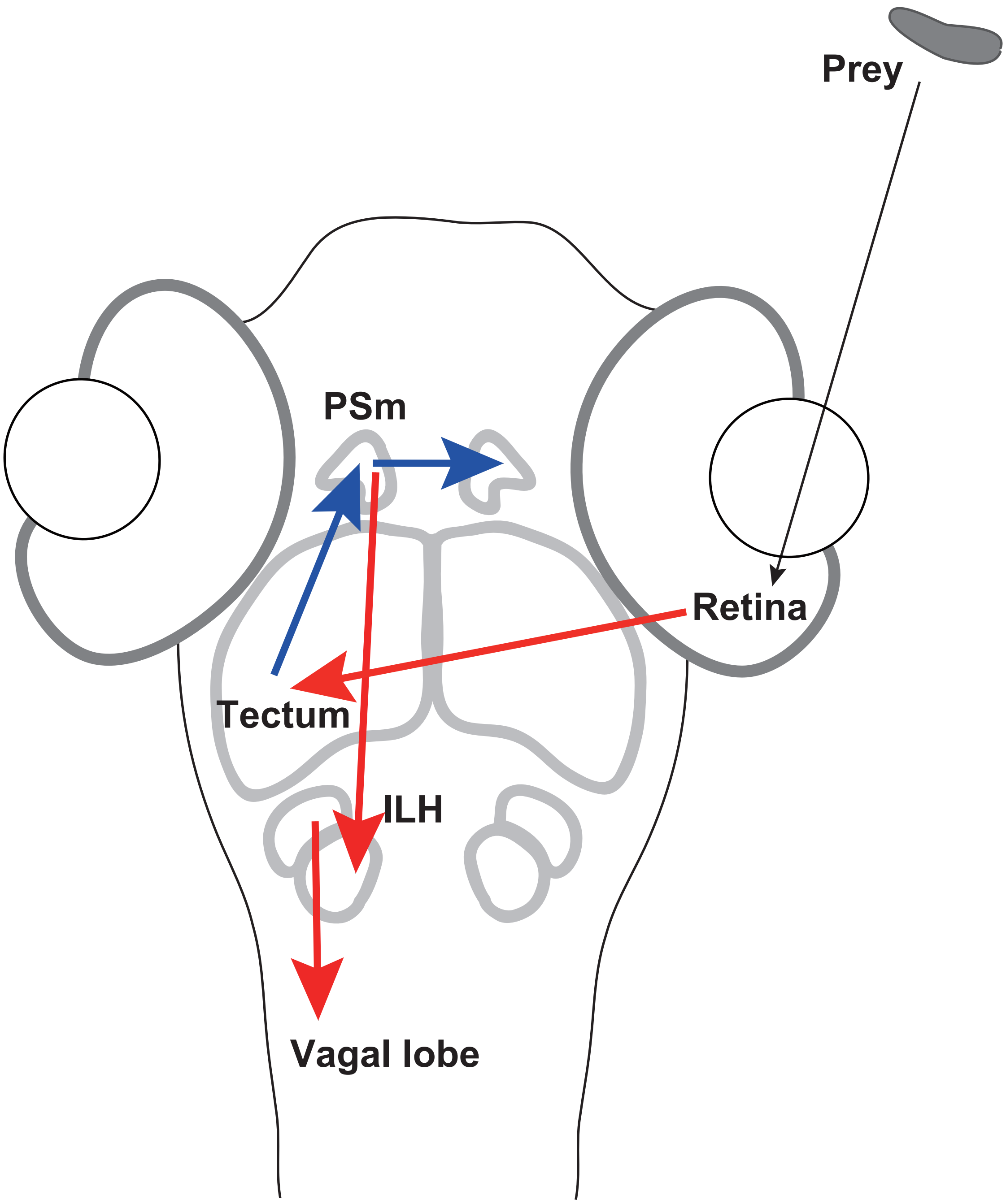

Supplementary Figure 14. Model of the neural circuits of prey capture

The sight of prey forms an image that is projected onto the retina. The majority of the retinal ganglion cells project to the optic tectum of the midbrain. The nucleus pretectalis superficialis pars magnocellularis (PSm)(gSAIzGFFM119B-labelled cells) presumably receives inputs from the optic tectum and then projects to the posterior part of the inferior lobes of the hypothalamus (ILH) (hspGFFDMC76A-labelled cells). The anterior part of the ILH projects through the dorsal area of the hindbrain (presumably, vagal lobe). Bilateral activation of the pretectum might be produced by the interconnection between the left and right pretectal areas. The red arrows indicate the axonal projections that are based on the morphological data shown in this study. The blue arrows indicate speculative connections. The projections are shown only on the left side of the schematic for clarity.
